# Supplementary material for: Characterizing cell-type spatial relationships across length scales in spatially resolved omics data
Source: Nat Commun. 2025 Jan 3;16:350. doi: 10.1038/s41467-024-55700-1 (PMC11699133; doi:10.1038/s41467-024-55700-1)
Supplement: Supplementary file 3 — Supplementary Software 1 [file 41467_2024_55700_MOESM3_ESM.zip › CRAWDAD/plots/seqfish/2_endothelium_tissue_grid.pdf]

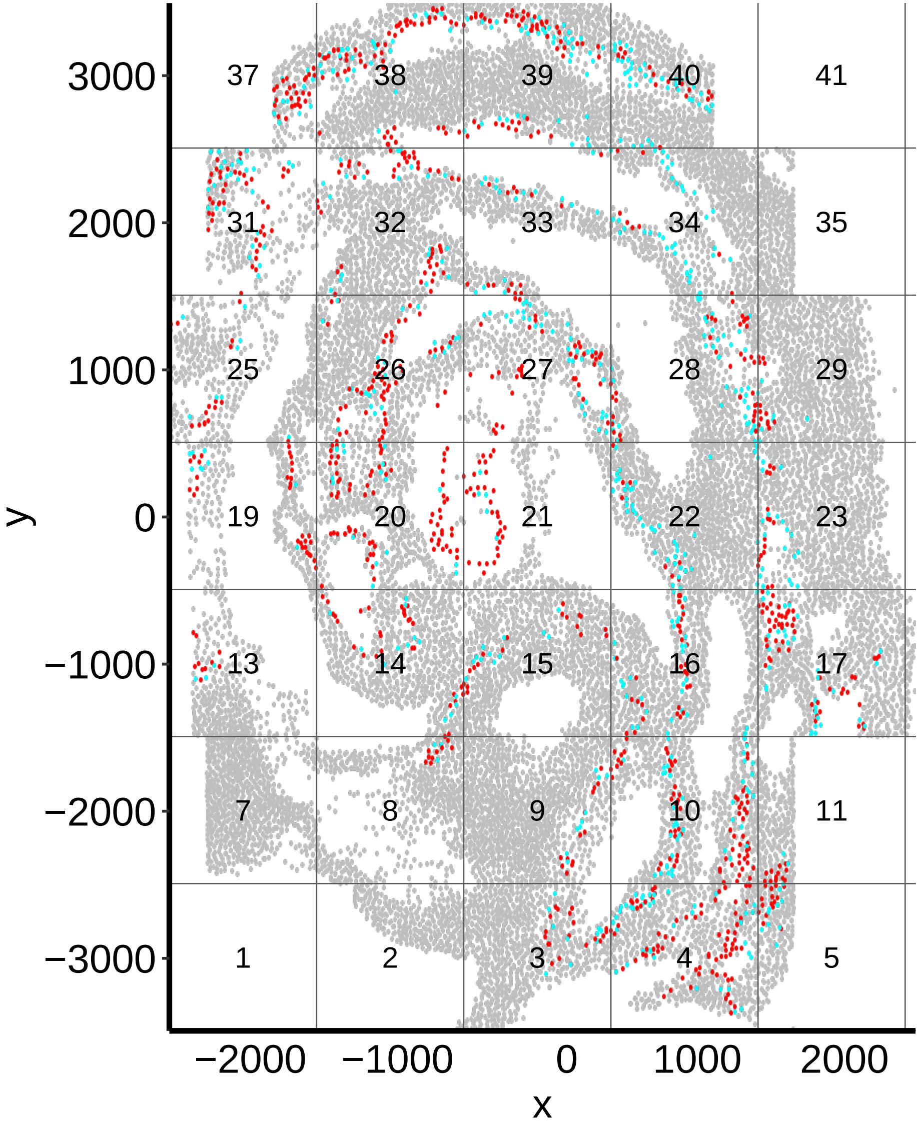

37
38
39
40
41
31
32
33
34
35
25
26
27
28
29
19
20
21
22
23
13
14
15
16
17
7
8
9
10
11
1
2
3
4
5
3000
2000
1000
0
−1000
−2000
−3000
y
−2000
−1000
0
1000
2000
x
